# Rapid Response High Temperature Oxygen Sensor Based on Titanium Doped Gallium Oxide

**DOI:** 10.1038/s41598-019-54136-8

**Published:** 2020-01-13

**Authors:** Sandeep Manandhar, Anil K. Battu, Arun Devaraj, V. Shutthanandan, S. Thevuthasan, C. V. Ramana

**Affiliations:** 1 Center for Advanced Materials Research CMR, University of Texas at El Paso, El Paso, Texas 79968 USA; 2 Environmental Molecular Sciences Laboratory, Pacific Northwest National Laboratory, Richland, Washington 99352 USA; 3 Physcial & Computational Sciences Directorate, Pacific Northwest National Laboratory, Richland, Washington 99352 USA

**Keywords:** Sensors and biosensors, Synthesis and processing

## Abstract

Real-time monitoring of combustion products and composition is critical to emission reduction and efficient energy production. The fuel efficiency in power plants and automobile engines can be dramatically improved by monitoring and controlling the combustion environment. However, the development of novel materials for survivability of oxygen sensors at extreme environments and demonstrated rapid response in chemical sensing is a major hindrance for further development in the field. Gallium oxide (Ga_2_O_3_), one among the wide band gap oxides, exhibit promising oxygen sensing properties in terms of reproducibility and long term stability. However, the oxygen sensors based on β-Ga_2_O_3_ and other existing materials lack in response time and stability at elevated temperatures. In this context, we demonstrate an approach to design materials based on Ti-doped Ga_2_O_3_, which exhibits a rapid response and excellent stability for oxygen sensing at elevated temperatures. We demonstrate that the nanocrystalline β-Ga_2_O_3_ films with 5% Ti significantly improves the response time (~20 times) while retaining the stability and repeatability in addition to enhancement in the sensitivity to oxygen. These extreme environment oxygen sensors with a rapid response time and sensitivity represent key advancement for integration into combustion systems for efficient energy conversion and emission reduction.

## Introduction

Recent attention to climate change and global warming has directed enormous global interest in reducing carbon dioxide (CO_2_) emissions to the environment. The major contributor to CO_2_ emission from human activities is from the energy conversion, especially the fossil fuel combustion for electricity, heat and transportation^1,2^. The strict regulation and mandates of governments to protect environments has also motivated the energy and transportation sectors to research in emission reduction and environmental protection. While transportation industry is trying to meet these stringent CO_2_ emission standards by reducing vehicle weight^3–5^, extensive research and development of new materials is needed to meet the weight reduction which is highly expensive and only a long-term solution. However, the emissions from fossil fuel combustion can be greatly reduced in both energy and transportation industry if internal engine combustion conditions are efficiently controlled. The optimization of combustion environment requires a reliable and rapid response chemical sensor to monitor the partial pressure of oxygen (pO_2_) during combustion. Hence, in recent years, the demand of fast and reliable oxygen sensing is rising exponentially^6,7^. No doubt that the earlier investigations resulted in a great success in fundamental understanding and development of commercialized thin film sensors based on metal oxides, such as SnO_2_ for domestic gas leak alarms, solid-electrolyte ZrO_2_ sensors for detecting oxygen concentration in automobile exhaust system, and TiO_2_-based lambda lean burn sensors^8,9^. However, addressing the poor stability, reliability, and slow response still remains a technological challenge. Wide band gap β-Ga_2_O_3_ has shown promise as a stable extreme environment chemical sensor^10–12^ due to excellent long term thermal stability (melting point ~1780 °C) against interfering gases and humidity^13^. Due to the high thermal stability of β-Ga_2_O_3_, the upper limit for operating temperature is ~1000 °C^6^. β-Ga_2_O_3_ is an n-type semiconductor at elevated temperatures and its conductivity is based on an oxygen deficiency of the crystal lattice. The resulting oxygen vacancies are ionized to form donors^14^. The material exhibits the same charge carrier mobility in the monocrystalline and in the polycrystalline state which means electron mobility is independent of grain boundaries^14^. It is also found that the sensors based on β-Ga_2_O_3_ films show high reproducibility and stability in the gas-sensitive electrical properties^6^. Also there has been some reports of β-Ga_2_O_3_ thin films operating as surface-control type sensors when exposed to a reducing gas below 900 °C^15^ and bulk type sensor when exposed to an oxygen above 900 °C^16^. Despite the advantages, response time (>1 min) for β-Ga_2_O_3_ based oxygen sensors (~60–200 s) are still inept for controlling efficient combustion^17,18^. In this context, in the present work, we demonstrate an approach based on selective doping metal ions into β-Ga_2_O_3_ to realize reliable and rapid response high-temperature oxygen sensors for operation in harsh environments.

As demonstrated and reported previously^11,19–24^, doping with carefully chosen metal ions can significantly alter the properties and electrical response of β-Ga_2_O_3_, making it suitable for high temperature chemical sensing applications. From an optical properties point of view, doping β-Ga_2_O_3_ with Sn, Cr, Cu, Ti, Mo, In, Fe, or W has been reported to induce changes in the optical absorption and band gap^11,19–24^. In literature, there has been some work in improving gas sensing performance of β-Ga_2_O_3_ by doping Ce, Sb, W and Zn^18^. The advantage of using dopants like Ce^4+^, Sb^5+^, W^6+^ is due to similar ionic radii which might allow the substitutional lattice sites resulting in lower sensor resistivity. However, the fastest response from these dopants was still ~1 min (60 s)^18^, which is unacceptable in managing combustion process efficient. Here in this paper, we report on an innovative approach to tailor the functionality of β-Ga_2_O_3_ by doping Ti to dramatically improve the response time of the oxygen sensor while retaining the thermal stability and reliability of β-Ga_2_O_3_ for high-temperature sensor applications. In the previous work, we report on the direct, functional relationship between the structural and optical properties of titanium (Ti)-incorporated Ga_2_O_3_ (GTO), where tailor-made materials for optical and photocatalytic applications are readily possible^19^. The hypothesis postulated based on the outcomes is that combining advantages of Ga- and Ti- based oxides through a selective doping of Ti into β-Ga_2_O_3_ and/or forming a Ga-Ti-O composite can enhance the sensor performance. The impetus is derived from the following considerations. Titanium, as a dopant, is attracted to oxygen and excess Ti will result in TiO_2_ phase. TiO_2_, a wide band gap semiconductor, has also been intensively studied as a key material for fundamental research and numerous technological applications because of its stability, non-toxicity, high abundance and low cost^25^. Furthermore, similar to β-Ga_2_O_3_, TiO_2_ is also an *n*-type semiconductor with a potential as a sensor^26–28^. It is noted that oxygen vacancies in transition metal oxides such as TiO_2_ are much more mobile than cations^29^, and it is reasonable to consider the movement of oxygen ions (or oxygen vacancies) as opposed to cations^30^. In addition, Shannon ionic radii^31^ of Ti^4+^ ionic radii in both octahedral and tetrahedral coordination closely matches with that of Ga^3+^. Hence, we propose and demonstrate designing more efficient materials for high-temperature oxygen sensing in harsh environments is readily possible by selectively doping Ti into β-Ga_2_O_3_.

## Results and Discussion

### Sensor performance and mechanism

**Figure 1:**
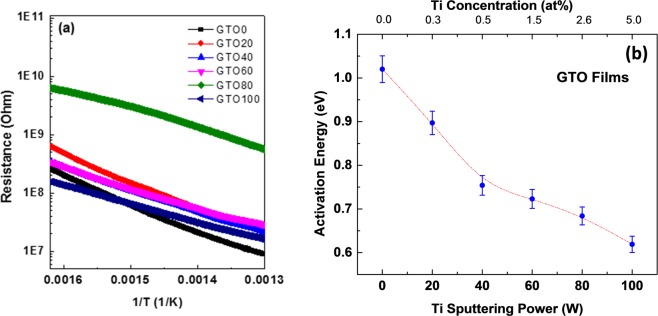
(**a**) Electrical data of GTO films. Arrhenius plots are shown for samples with variable Ti content. (**b**) Activation energy of GTO films. Continuous decrease in E_A_ values with Ti incorporation into Ga_2_O_3_ can be noted.

The temperature dependent electrical data, which serves the baseline characteristics for sensor applications, of GTO samples are shown in Fig. 1. It is generally accepted that barrier formation between grains is responsible for the sensor conductivity and that these barriers have a Schottky-type nature^32^. Therefore, as a first step, determination of activation energy is quite important for utilization of GTO for sensor applications. The temperature-dependent electrical data (Fig. 1a; Arrhenius plots) of GTO samples were analyzed to obtain the activation energy of the films using the relation:

1 $$R={R}_{\infty }{e}^{(-\frac{{E}_{A}}{kT})}$$

where R_∞_ is the resistance at infinite temperature, E_A_ is the activation energy, k is the Boltzmann constant, T is the absolute temperature, and R is the resistance. The E_A_ values determined are presented in Fig. 1b, where the effect of Ti content on the E_A_ values is shown. It can be seen that the E_A_ continuously decrease with increasing Ti in Ga_2_O_3_. This observation is imperative since the E_A_ reduction is expected to contribute the enhanced sensitivity. The E_A_ value for intrinsic Ga_2_O_3_ films was 1.02 eV, which reduces to a final value of 0.62 eV when the Ti concentration is ~5 at%. Significant reduction in E_A_ is an indication that thermal energy required for oxygen detection becomes smaller by the incorporation of Ti-ions into Ga_2_O_3_.

**Figure 2:**
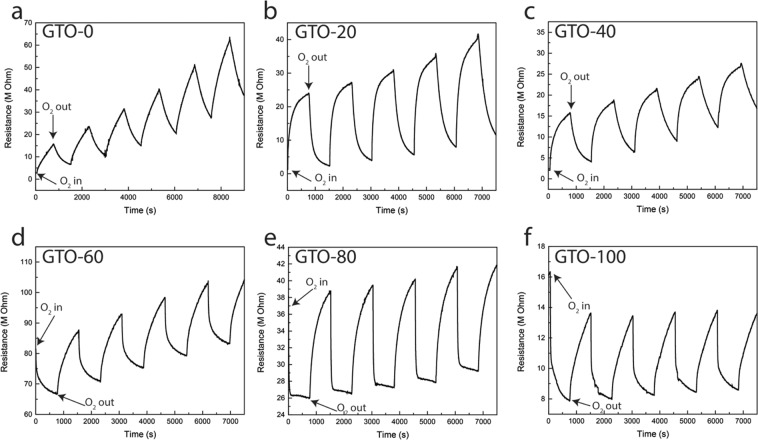
Gas sensing performance test. (**a**) GTO-0. (**b**) GTO-20. (**c**) GTO-40. (**d**) GTO-60. (**e**) GTO-80. (**f**) GTO-100.

The oxygen sensor response data of GTO films are shown in Fig. 2. The performance characteristics were evaluated at 700 °C in terms of initial sensitivity and response time. The data shown are obtained in the experiments conducted under different pO_2_ (8–40 Pa) in cyclic periods of pure Ar flow for a constant time followed by Ar/O_2_ flow for the same time period. The repeatability and stability of the sensors was evaluated (Fig. 2) at 700 °C for several hours as a function of pO_2_. All of the GTO films demonstrated the stability and repeatability for the extended duration of the tests (≥100 h). No discontinuity or breakdown was observed over the range of pO_2_ examined. Intrinsic Ga_2_O_3_ response also presented changes in the resistance value under Ar exposure, corresponding the polarization effects caused by the ionic conductivity of the films, and the constant increment on the base resistance is attributed to the grain boundary capacitance of the films^33^. As shown Fig. 2, it can be observed that GTO-0, GTO-20, and GTO-40 responded by the increase in resistance when oxygen is introduced. This response of pure Ga_2_O_3_ has been previously observed in the literature^17^. However, the changes in response behavior becomes evident when the Ti content becomes appreciable. For Ti a concentration of ≥1.5 at%, the sensor response to oxygen flips and resistance now decreases after oxygen is introduced and vice-versa. This remarkable behavior observed for GTO films under pO_2_ is totally different and has not been achieved or reported earlier for intrinsic Ga_2_O_3_. Also, the changes are rapid; the response behavior in GTO films is rather instantaneous and occur rapidly upon oxygen release into the argon stream. However, when the oxygen was cut off, the reversal to original resistance takes bit of a longer time. This phenomenon confirms that out-diffusion of oxygen is relatively slow. To ensure reproducibility, interval between successive measurements is set to ~10 min.

**Figure 3:**
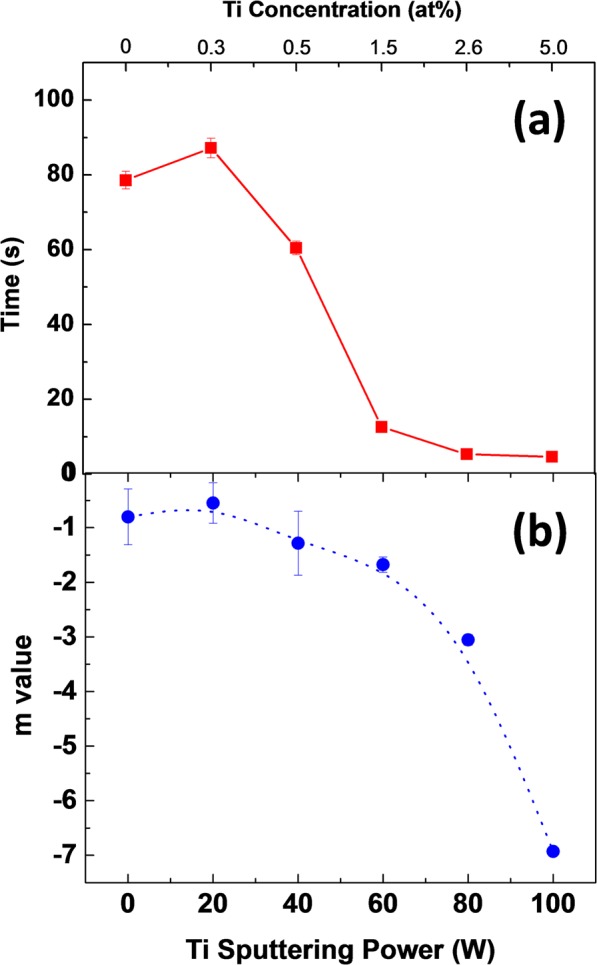
Oxygen sensor performance of characteristic values of GTO films. (**a**) Response time. (**b**) m value determined from functional electrical characteristics of GTO.

The response time GTO sensors are determined, using the standard procedures, from the sensor characteristics at various pO_2_ (Fig. 2). The response time for intrinsic Ga_2_O_3_ (GTO-0) is 80 ± 10 s, whereas for the films deposited with 5 at% of Ti-doped Ga_2_O_3_ exhibits a rapid response time of only 4 ± 1 s. This is a remarkable result and demonstrates the effect of Ti into Ga_2_O_3_ to promote oxygen sensor performance. The sensor response data is presented in Fig. 3a, where the results indicate that increasing Ti content in Ga_2_O_3_ decreases the response time i.e., improves the sensor performance. The results indicate that the 5 at% Ti doped Ga_2_O_3_ is nearly ~20 times faster as compared to intrinsic Ga_2_O_3_. Note that the response time can be influenced by the surface reactions rate or/and the diffusion rate. When pO_2_ is increased, the oxygen molecules adsorbed at the surface split and occupy the vacancies near the surface. A diffusion process of oxygen inside the film, across the grain boundaries, will reestablish equilibrium with the gas phase^15^.

The materials that respond to change in pO_2_ in the upper-temperature range (700 °C and above) are reflecting the equilibria between the atmosphere and their bulk stoichiometry. In general, the relationship between oxygen partial pressure and the electrical conductivity of the mixed valence oxide sensor may be represented^34^ by:

2 $$\sigma =A\ast \exp [\,-\,{E}_{A}/kT]/p{O}_{2}^{1/m}$$

where A is a constant, pO_2_ is the partial pressure of oxygen, and m is a parameter determined by the carriers (*n* or *p* type) and defects in the oxide^34,35^ while other parameters were defined already in Eq. (1) The value of |m| is the sensitivity of the sensor; higher the |m| value, higher the sensitivity and vice versa^35^. The electrical data at various pO_2_ were used to calculate the m-values, which are presented in Fig. 3b. In intrinsic Ga_2_O_3_ films (GTO-0), the m-value is −0.8, which is quite higher than theoretical value (at 1000 °C) of −4 as presented in the literature^13,17^. However, the Ti doping in Ga_2_O_3_ shows the reduction of m value up to −6.92 for GTO-100, i.e., Ti concentration of 5 at%. From Eq. 2, it is clear that the lower value of m value corresponds to a higher sensitivity of sensor in terms of its response to changes in pO_2_. Also, lower value of E_A_ corresponds to lower sensitivity to the temperature fluctuations. The value of m for pure Ga_2_O_3_ (GTO-0) and highest Ti concentration of 5 at% (GTO-100) are −0.8 and −6.92, respectively, where lower m value of GTO-100 demonstrates a better oxygen sensitivity. Similarly, GTO-100 samples with lowest E_A_ (0.62 eV) implies precise measurements of pO_2_ when employed for oxygen sensing.

### Structure

**Figure 4:**
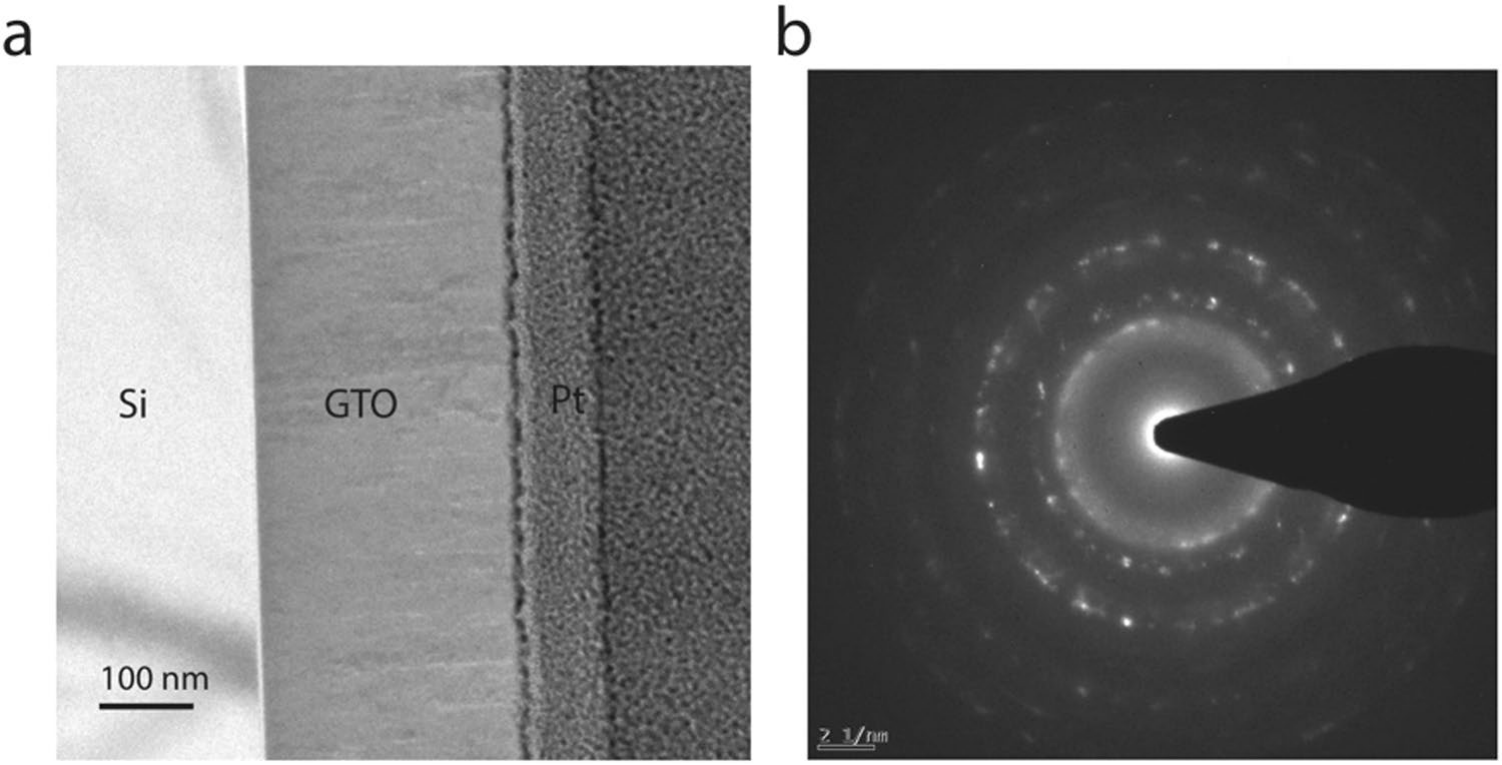
TEM and Selected Area Electron Diffraction (SAED) paired image of GTO-100 (5% Ti at%). (**a**) Bright-field transmission electron micrograph of a GTO-100. (**b**) SAED of the GTO-100 layers demonstrates ring pattern.

In order to better account for the observed sensor performance and to understand the structural changes with Ti, Transmission Electron Microscopy (TEM) analysis has been performed on GTO-100. The data obtained is shown in Fig. 4. The changes in microstructure due to doping is evident in Fig. 4. Bright-field TEM images reveal multiple layers of the samples indicating a Ga-Ti-O nanocomposite on Si. The top two layers are the Pt deposited using electron beam and ion beam subsequently during FIB lift out process (Fig. 4a). The ring patterns from the selected area electron diffraction (SAED) (Fig. 4b) confirms multiple phases in the sample, where the strongest peaks can be indexed to β-Ga_2_O_3_ but weaker intensity peaks are probably mixed with TiO_2_ (anatase). This overlap of multiple rings makes it impossible to confirm TiO_2_ phases accurately but confirms the crystallinity of the nanocomposite even with highest Ti doping (5% Ti at%) in β-Ga_2_O_3_.

### Surface and Interface Chemistry

**Figure 5:**
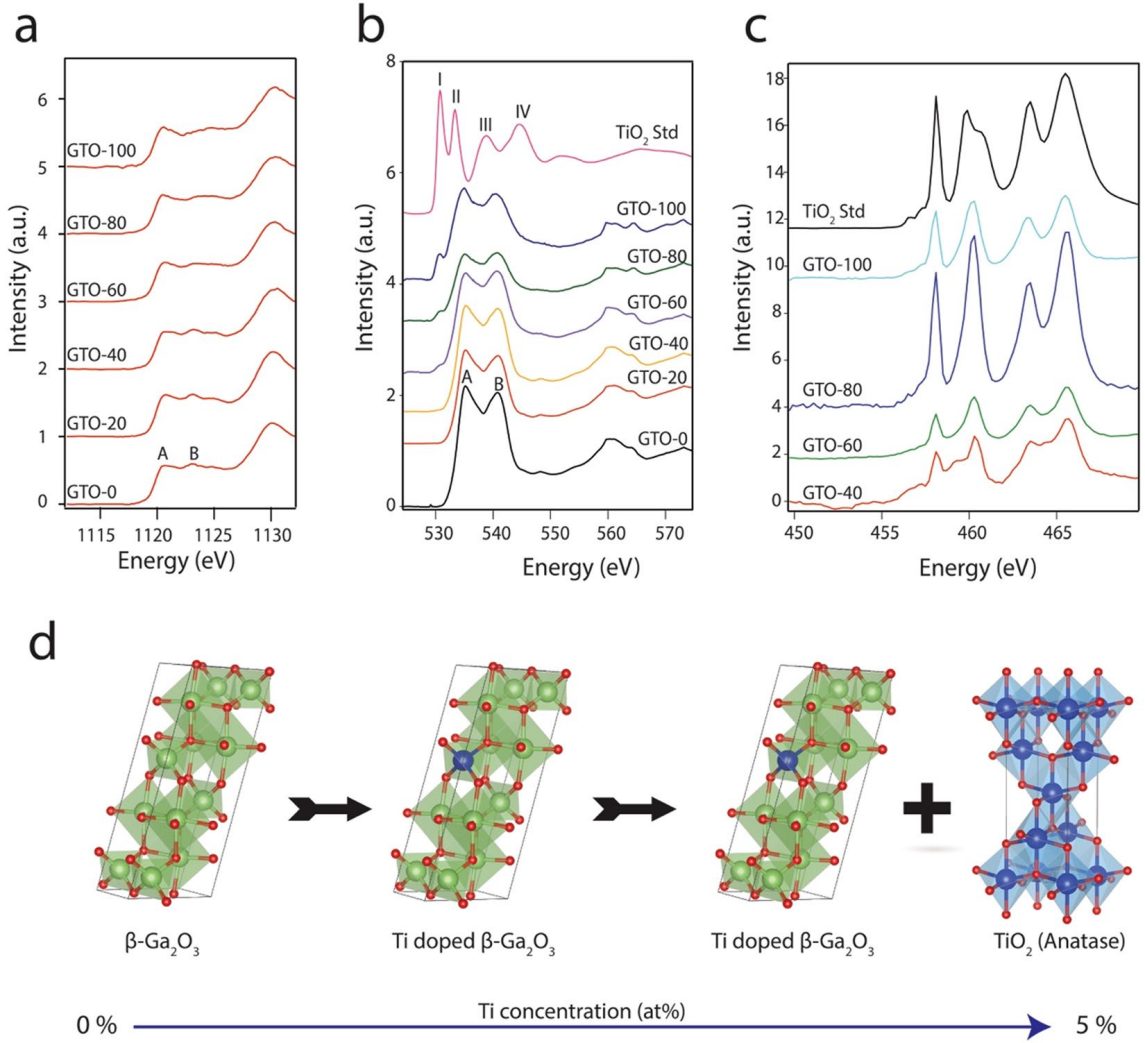
XANES spectra of Ga L edge, O K-edge, and Ti L-edge GTO films deposited with variable Ti-concentration. (**a**) Ga L-edge. (**b**) O K-edge. (**c**) Ti L-edge. (**d**) Illustration of Ti doping in Ga_2_O_3_ matrix.

To probe the chemical changes and better understand the dopant Ti chemistry in β-Ga_2_O_3_ and identify the TiO_2_ phase which was inconclusive in TEM, X-ray Absorption Near Edge Structure (XANES) was employed. In XANES, the fine structure observed above the edge, so-called white line, reflects the local site symmetry around the X-ray absorbing atom, the analysis of this region is for the fingerprint of the elements. It is well known that β-Ga_2_O_3_ has a spinel structure, containing Ga atoms in both tetrahedral and octahedral sites^36,37^. The Ga L-edge spectra shown in Fig. 5a is due to excitation of electrons in the 2p_3/2_ orbital of Ga, leading to sharp increase in absorption of Ga-containing materials at 1116.4 eV^38^. However, the edge for Ga in Ga_2_O_3_ is shifted to 1120 eV due to valence dependency which causes edges to shift^39^. Therefore, the first peak labeled A (or edge) in Ga L edge spectrum is assigned to Ga atoms in octahedral sites while subsequent peak labeled B is assigned to Ga in tetrahedral sites^37^. As the Ti concentration is steadily increased the signature peak of tetrahedral Ga atoms is diminished, which is presumably due to the replacement of Ga atoms in tetrahedral sites by Ti atoms.

In Fig. 5b, O K-edge spectra of GTO samples is shown along with the TiO_2_ standard. The O K-edge spectra for TiO_2_ standard matches signature features of anatase^40^ which confirms the TiO_2_ phase. In anatase (TiO_2_ standard), first two peaks at low energy (I and II) is due to dipole transitions to band states of t_2g_-e_g_ symmetry separated by the “ligand-field splitting” of O 2p states hybridized with the Ti 3d states localized at the Ti sites^41^. The next two peaks (III and IV) in TiO_2_ standard is due to transitions to oxygen 2p hybridized with Ti 4p states of respective local symmetry *b*_3_ and *e* in their octahedral environment^40^. The O K-edge XANES spectrum of the Ga_2_O_3_ (GTO-0) nanostructure sample is quite different from that of the TiO_2_ standard. For the GTO-0 sample, XANES spectrum exhibits two main peaks at ~535 eV and ~542 eV (A and B. respectively), similar to that of the β-Ga_2_O_3_^42^. Also from the crystal structure, it can be deduced that oxygen also resides in both octahedral and tetrahedral sites. Here, the difference of the O K-edge XANES spectra shown in Fig. 5b between the two samples (TiO_2_ standard and GTO-0) is the pre-edge feature due to Ti doping after GTO-60. This pre-edge (~530 eV) arise from the existence of an energy state in the band gap. In Fig. 5c, Ti L-edge XANES spectra of GTO samples are shown, but there was no titanium detected by XANES below for GTO-20. For anatase (TiO_2_) standard and all GTO samples, two sets of peaks are observed which are separated by ~6 eV. This splitting is due to core-hole spin-orbit splitting of the 2p levels^41,43^. It is believed that the structure of these two sets of peaks is from splitting of the d-derived final states into t_2g_- and e_g_-like levels^44,45^. This two set of the peak is also seen in GTO samples where titanium is above the detection limit in XANES. This XANES study basically suggests Ti doping in β-Ga_2_O_3_ substitutes at tetrahedral position but at higher concentration Ti phase separates in TiO_2_ (anatase) as shown in illustration in Fig. 5d.

**Figure 6:**
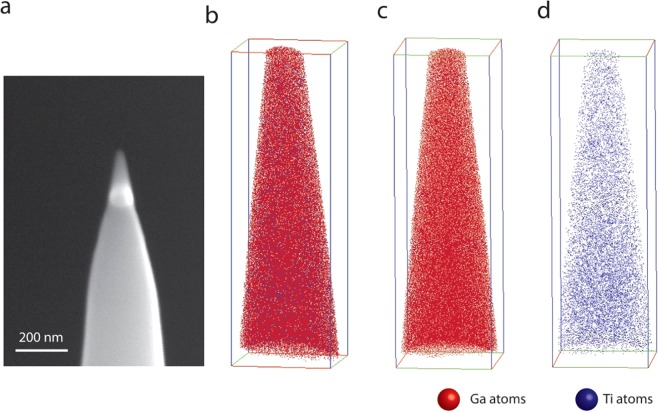
APT reconstruction of GTO-100 samples. (**a**) Sample tip as prepared after FIB annular milling. (**b**) All-atomic view of APT reconstruction showing Ga in red and Ti in blue. (**c**) Atomic view of only Ga atoms. (**d**) Atomic view of only Ti atoms.

The quality and atomic scale defects and clustering were further examined by atom probe tomography (APT) of the sample with highest Ti concentration, i.e., 5 at% (GTO-100). Specifically, APT allows us to verify dopant clustering (if any) at the highest concentration. The APT specimens were lifted out using focused ion beam (FIB) using Omni Probe and mounted on the micro-post for annular milling. The APT reconstructions of GTO-100 specimen are shown in Fig. 6 with Ga atoms in red and Ti in blue. The APT reconstruction shows a 37.78 × 35.98 × 119.1 nm3 volume. From the APT reconstruction, Ti is evenly distributed through the entire film thickness and no noticeable cluster formation of Ti atoms even for the highest Ti concentration. Thus, the XANES and APT data accounts for the structural and chemical quality of the GTO films.

## Conclusions

An approach based on selective doping of Ti into β-Ga_2_O_3_ is demonstrated to realize reliable and rapid response high-temperature oxygen sensors for utilization in power plants and transportation systems. In Ti doped β-Ga_2_O_3,_ a rapid response time of ~ 4 s is noted for oxygen sensor operating at 700 °C. This is remarkably high (~20 times) compared to the intrinsic Ga_2_O_3_ or existing other oxygen sensors. Ti-doped β-Ga_2_O_3_ exhibits lowest activation energy, low *m*-value, good recovery time, and excellent repeatability. The structural and chemical analyses indicate the role of Ti in β-Ga_2_O_3_ to promote the sensor performance. We believe that there may be further options available to further tune and improve the sensor performance while the underlying science and mechanisms may be applicable to a large class of “doped nanostructured oxides for sensor applications.”

## Methods

### Sample preparation

Gallium oxide and Ti-incorporated Ga_2_O_3_ films were deposited onto silicon (Si) and quartz with thin film Pt interdigitated finger electrodes with 100 μm lines substrates by radio-frequency magnetron sputtering. Co-sputtering of the Ga_2_O_3_ (99.999%) and Ti (99.95%) targets (2 in. diameter; Plasmaterials Inc.) was performed to produce GTO films. All the substrates were thoroughly cleaned and dried with nitrogen. The Ga_2_O_3_-and Ti targets were placed on 2-in. sputter guns, which were placed at 8 cm from the substrate. The base pressure was ~10^−6^ Torr. A sputtering power of 25 W was initially applied to the targets while introducing high-purity argon (Ar) into the chamber to ignite the plasma. Once the plasma was ignited, the sputtering power was increased to the desired or set values for Ga_2_O_3_ and Ti targets, respectively. A constant sputtering power of 100 W was maintained for the Ga_2_O_3_ target. The Ti-target sputtering power was varied in the range of 0–100 W to vary the Ti concentration (x) in the films. For clarity purposes, the sample identification is made with the sputtering power employed for deposition. Thus, the samples with variable Ti content are named GTO-0, GTO-20, GTO-40, GTO-60, GTO-80, and GTO-100, respectively, where the end numbers (0–100) represent sputtering power applied to the Ti target. The flows of argon and oxygen were controlled using MKS mass flow meters. Before each deposition, the Ga_2_O_3_ target was pre-sputtered for 10 min with the shutter above the gun closed. The samples were deposited at a substrate temperature (T_s_) of 500 °C, which is optimum for producing nanocrystalline, β-phase Ga_2_O_3_ films^19,46^. The substrates were heated by resistive heating, and the desired T_s_ was controlled by an Athena X25 controller. The time of deposition was kept constant at 3 hours. The doping of titanium on gallium oxide is done by substitutional of gallium site with titanium and creates gallium vacancies as shown below by Kroger-Vink notation:

$$3Ti{O}_{2}\,\mathop{\longrightarrow }\limits^{G{a}_{2}{O}_{3}}\,3T{{i}_{Ga}}^{\cdot }+6{{O}_{O}}^{X}+{V\prime\prime\prime }_{Ga}$$

The solubility of titanium in Ga_2_O_3_ can be simply explained by the comparing its ionic radii, i.e., Ga^3+^ has ionic radii of 0.047 nm and 0.062 nm for the coordination number of 4 and 6 while Ti^4+^ has ionic radii of 0.042 nm and 0.06 nm for the coordination number of 4 and 6^35,47^. They (Ga and Ti) also have similar electronegativity (1.6 Pau and 1.5 Pau respectively)^48^.

### X-ray absorption near edge structure (XANES)

The XANES experiments were carried out at beamline 6.3.1 of Advanced Light Source (ALS) at Lawrence Berkeley National Laboratory (LBNL). Beamline 6.3.1 utilizes a variable line space (VLS) monochromator on a bend magnet source to provide photons with energies from 100 to 2200 eV into a multi-purpose end-station that is like the end-station on Beamline 6.3.2^49^. Reference Ti L-edge, Mg K-edge, and O K-edge spectra were also collected from TiO_2_ and MgO standards for calibration for different gratings used. Spectra were collected at room temperature in the total electron yield (TEY) mode with the signal obtained from the sample drain current. No substantial charging problems were detected. Ga L-edge, O K-edge, and Ti L-edge were collected for all samples.

### Atom probe tomography (APT)

APT samples were also prepared using the FEI Quanta dual beam FIB. The APT specimen preparation method by site-specific FIB lift-out and annular milling^50,51^. A CAMECA LEAP 4000XHR system equipped with pulsed UV laser (355 nm wavelength) was used to perform APT experiments using 40pJ laser pulse energy at the 100 kHz frequency with specimen temperature of 40 K. The APT results were reconstructed and analyzed using Interactive Visualization and Analysis Software (IVAS) 3.6.8 using standard reconstruction procedure^51^.

### Transmission electron microscopy (TEM)

TEM samples were prepared using lamella lift out procedure using dual beam FIB (FEI Helios) and mounted on copper half grids. The TEM was done using FEI Tecnai TEM using field emission gun operating with an accelerating voltage of 200 kV.

### Oxygen sensor testing and evaluation

The sensor performance was evaluated at a temperature of 700 °C; the electrical measurements were recorded using Keithley 6514 electrometer, and the input current (10 nA) was supplied via a Keithley 220 Programmable Current Source. Two gas tanks were employed for this investigation, a 99.99% Ar as the baseline gas, and a 99.99% O_2_ as the analyte gas; both gasses were controlled using an MKS mass flow controller to achieve different partial pressures of oxygen. All the sensor performance responses were recorded as a function of Ti sputtering power and Ti concentration and feature such sensitivity and time response are presented.

The activation energy was calculated using Eq. 1. The experiments were conducted at different partial pressures. Each partial pressure was subjected to 6 cycles (N = 6) for repeatability. Each and every fitting procedure is carried out until the best fit (R is greater than 0.9 or better) is achieved. The R values were obtained from fitting for each partial pressure along with the standard deviation which was used to calculate the final value of activation energy. The error of fitting is as reflected in the data graphs presented, where the error estimation and statistical standard deviation is also considered.

## Data Availability

The necessary data required is included in the manuscript. The data available in the literature has been cited as needed and appropriate.
